# Efficacy and Safety of Lobeglitazone Monotherapy in Patients with Type 2 Diabetes Mellitus over 24-Weeks: A Multicenter, Randomized, Double-Blind, Parallel-Group, Placebo Controlled Trial

**DOI:** 10.1371/journal.pone.0092843

**Published:** 2014-04-15

**Authors:** Sin Gon Kim, Doo Man Kim, Jeong-Taek Woo, Hak Chul Jang, Choon Hee Chung, Kyung Soo Ko, Jeong Hyun Park, Yong Soo Park, Sang Jin Kim, Dong Seop Choi

**Affiliations:** 1 Department of Internal Medicine, Korea University Anam Hospital, Seoul, Korea; 2 Department of Internal Medicine, Hallym University Kangdong Sacred Heart Hospital, Seoul, Korea; 3 Department of Internal Medicine, Kyung Hee University Hospital, Seoul, Korea; 4 Department of Internal Medicine, Seoul National University Bundang Hospital, Seongnam, Korea; 5 Department of Internal Medicine, Wonju Severance Christian Hospital, Wonju, Korea; 6 Department of Internal Medicine, Inje University Sanggye Paik Hospital, Seoul, Korea; 7 Department of Internal Medicine, Inje University Pusan Paik Hospital, Pusan, Korea; 8 Department of Internal Medicine, Hanyang University Guri Hospital, Guri, Korea; 9 Department of Internal Medicine, Soon Chun Hyang University Cheonan Hospital, Cheonan, Korea; Johns Hopkins Bloomberg School of Public Health, United States of America

## Abstract

**Objective:**

The aim of this study was to assess the glucose-lowering and lipid-modifying effects, and safety profile of lobeglitazone, a novel peroxisome proliferator-activated receptor- γ agonist, compared to placebo as a monotherapy in patients with type 2 diabetes.

**Research Design and Methods:**

In this 24-week, multicenter, randomized, double-blind, parallel-group, placebo controlled study, 173 patients were randomly assigned (a 2∶1 ratio) to lobeglitazone 0.5 mg (n = 115) or matching placebo (n = 58) orally once daily. The primary endpoint was the change in glycated hemoglobin (HbA1c) from baseline to the end of treatment. The secondary endpoints included various glycemic parameters, lipid parameters and safety profile (ClinicalTrials.gov number NCT01001611).

**Results:**

At 24 weeks, a significant reduction in HbA1c was observed with lobeglitazone versus placebo (−0.44% vs 0.16%, mean difference −0.6%, p<0.0001). The goal of HbA1c <7% was achieved significantly more in the lobeglitazone group compared to the placebo group (44% vs 12%, p<0.0001). Markers of insulin resistance were also improved in the lobeglitazone group. In addition, lobeglitazone treatment significantly improved triglycerides, high density lipoprotein cholesterol, small dense low density lipoprotein cholesterol, free fatty acid, and apolipoprotein-B/CIII compared to placebo (p<0.01, respectively). More weight gain was observed in the lobeglitazone group than the placebo group (0.89 kg vs – 0.63 kg, mean difference 1.52 kg, p<0.0001). The safety profile was comparable between the two groups and lobeglitazone was well tolerated.

**Conclusions:**

Lobeglitazone 0.5 mg showed a favorable balance in the efficacy and safety profile. The results support a potential role of lobeglitazone in treating type 2 diabetes.

**Trial Registration:**

Clinicaltrials.gov NCT01001611

## Introduction

Thiazolidinediones (TZDs), peroxisome proliferator-activated receptor (PPAR)-γ agonists, are the first drugs that improve insulin sensitivity in skeletal muscle and reduce hepatic glucose production in patients with type 2 diabetes mellitus (T2DM) [1]. They do not increase the risk of hypoglycemia and are more durable in controlling hyperglycemia than sulfonylureas and metformin [2]. Furthermore, pioglitazone has a beneficial effect on the lipid profile in patients with T2DM [3]. The PROspective pioglitAzone Clinical Trial In macroVascular Events (PROactive) trial showed a benefit on major cardiovascular events as a secondary outcome in patients with a prior cardiovascular event or with multiple risk factors for cardiovascular disease (CVD) [4].

However, TZDs may have clinically significant adverse effects (AEs), such as body-weight gain, fluid retention, congestive heart failure, bone fractures, increased risk of myocardial infarction, and possibly bladder cancer [5]. Owing to concerns of increased myocardial infarction risk, rosiglitazone is no longer widely available [6] and, due to concerns of its possible association with bladder cancer [7], use of pioglitazone has been suspended in some European countries including France. Therefore, there is a need to develop more effective and safe antidiabetic drugs targeting PPAR-γ [8].

Lobeglitazone (CKD-501; Chong Kun Dang Pharmaceutical Corp., Seoul, Korea) is a novel PPAR-γ agonist with substituted pyrimidine derivatives containing TZD **(Information S1)**. Lobeglitazone showed more potent activity than the reference compounds (i.e. pioglitazone and rosiglitazone) in both *in vitro* and *in vivo* studies [9], [10]. Therefore, lobeglitazone is expected to improve insulin sensitivity, and glucose and blood lipid profiles with a lower effective dose.

In a phase I trial, lobeglitazone was well tolerated up to 4 mg and the pharmacokinetic (PK) properties after a once-daily dose of lobeglitazone, treated for 7 days, were comparable to the single-dose PK properties [11]. Another clinical trial also demonstrated no statistically or clinically meaningful PK interactions as co-administration of lobeglitazone (0.5 mg/day) and metformin (1000 mg/day) during 5 days of treatment in healthy volunteers [12].

However, the efficacy and safety of lobeglitazone have not been established in a clinical trial of patients with T2DM. Therefore, the aim of this study was to assess the glucose-lowering and lipid-modifying effects, as well as the safety profile of lobeglitazone, compared to placebo as a monotherapy in patients with T2DM.

## Methods

### Study patients

Patients had to meet all the following inclusion criteria: age 18–80 years, T2DM diagnosed at least 3 months earlier, glycated hemoglobin (HbA1c) 6.5–9% at screening test if medication with oral hypoglycemic agents (OHAs) had been stopped less than 3 months ago or HbA1c 7–10% at screening test if patients were drug naïve or had ceased medications with OHAs more than 3 months previously, body mass index (BMI) between 21 kg/m^2^ and 40 kg/m^2^, and fasting serum C-peptide level exceeding 1.0 ng/mL.

The major exclusion criteria included fasting plasma glucose level over 250 mg/dL, triglyceride level over 500 mg/dL, treatment with insulin or TZDs within 60 days, uncontrollable hypertension, history of myocardial infarction, heart failure (New York Heart Association class III or IV), cerebral infarction, cerebral hemorrhage or unstable angina within 6 months, severe hepatic dysfunction, severe renal dysfunction, anemia for any reason, uncontrolled other disease or diabetic complications, concomitant use of drug(s) having severe drug interaction with the investigational drug, and a history of cancer within 5 years. Medication dosages of lipid-lowering drugs or oral contraceptives were maintained throughout the study.

### Ethics statement

Witten informed consent was obtained from all the patients before participation, and this study was approved by each study center's institutional review boards. The full names of each Institutional review boards were listed as follows:

Korea University Anam Hospital Institutional Review Board, Seoul National University Bundang Hospital Institutional Review Board, Kyung Hee University Hospital Institutional Review Board, Hallym University Kangdong Sacred Heart Hospital Institutional Review Board, Wonju Severance Christian Hospital Institutional Review Board, Inje University Sanggye Paik Hospital Institutional Review Board, Inje University Busan Paik Hospital Institutional Review Board, Hanyang University Guri Hospital Institutional Review Board and Soon Chun Hyang University Hospital Cheonan Institutional Review Board.

### Study design

This 24-week, multicenter, randomized, double-blind, parallel-group, placebo control, therapeutic confirmatory study was conducted at nine centers in South Korea between 2009 and 2011. The study consisted of a single-blind, 2-week placebo run-in period if patients were drug naïve or had stopped taking OHAs more than 3 months previously, and an additional 4-week wash out period before the run-in period if patients had stopped taking OHAs less than 3 months previously. Patients were randomized in a 2∶1 ratio to receive double-blind treatment with 0.5 mg lobeglitazone or matching placebo for 24 weeks. Patients completing the study treatment phase were eligible for participation in a 28-week, open-label extension phase to evaluate the long-term safety, during which patients on lobeglitazone continued on the same dose, whereas patients in the placebo group were switched to lobeglitazone (data not presented).

The study medications were administered orally once daily in the morning (irrespective of the time of breakfast). During the treatment period, if fasting plasma glucose (FPG) exceeded pre-specified levels, rescue medication (metformin) was introduced. Randomization was achieved by center using restricted block randomization to ensure equal distribution. The randomization numbers of the patients were generated by the sponsor and provided in sequentially numbered, sealed, opaque envelopes. Double-blinding was maintained using identical lobeglitazone and placebo tablets. All patients received diet and lifestyle counseling with a written educational material.

Lobeglitazone dose selection was based on a phase II study performed in patients with T2DM. In an 8-week, randomized, double-blind, parallel-group, placebo-controlled, dose-ranging study, lobeglitazone administered at doses of 0.5, 1, or 2 mg once daily in patients with the same inclusion and exclusion criteria of this study. A total of 214 patients were randomly assigned to 0.5 mg lobeglitazone (n = 55), 1 mg lobeglitazone (n = 54), 2 mg lobeglitazone (n = 50), or matching placebo (n = 55). FPG as a primary endpoint was decreased by 20.65 mg/dL (0.5 mg lobeglitazone), 23.38 mg/dL (1 mg lobeglitazone), and 33.69 mg/dL (2 mg lobeglitazone), and this decrement was statistically significant compared to placebo, respectively. As a result, the minimum effective dose was determined to 0.5 mg lobeglitazone by the Williams test. The frequency of adverse events was comparable between groups except edema (0% at 0.5 mg, 5.8% at 1 mg, and 14.3% at 2 mg). Due to the increasing rate of edema in the higher doses, Korea Food and Drug Administration (KFDA) recommended that the minimum effective dose is used for further studies. Accordingly, in this study, we assessed the efficacy and safety of lobeglitazone 0.5 mg once daily.

### Study assessments

The primary endpoint was the change in HbA1c from baseline to the end of treatment. The secondary endpoints included changes from baseline in various glycemic parameters (HbA1c target achievement rate (HbA1c <7%), FPG, homeostasis model assessment of insulin resistance (HOMA-IR), homeostasis model assessment of β-cell function (HOMA-β), lipid parameters (total cholesterol, triglycerides, low density lipoprotein-cholesterol (LDL-C), high density lipoprotein-cholesterol (HDL-C), small dense LDL-C, free fatty acid (FFA), and apolipoprotein (Apo) AI/B/CIII). During the 24-week treatment period, patients visited the clinic at baseline and weeks 4, 10, 16, and 24, at which fasting blood samples were taken for assessment. Laboratory analyses for the primary and secondary endpoints were done by a central laboratory (Seoul Clinical Laboratories, Seoul, Korea). A1C levels were determined using turbidimetric inhibition immunoassay (Cobas Integra 400 Plus testing system; Roche Diagnostics, Indianapolis, IN, USA). Plasma glucose, triglycerides, total cholesterol, HDL-C and LDL-C were determined using enzymatic colorimetic assays (reagents obtained from Roche Diagnostics, Indianapolis, IN, USA), and FFA levels were determined using enzymatic colorimetic assays (reagents obtained from Shinyang Diagnostics, Seoul, Korea). Apo-A1 and Apo-B levels were determined using immunoturbidimetric assays (reagents obtained from Roche Diagnostics, Indianapolis, IN, USA), and Apo CIII were determined using immunoturbidimetric assays (reagents obtained from Nittobo Medical, Tokyo, Japan). Serum small dense LDL cholesterol levels were determined using polyacrylamide gel electrophoresis assays (Lipoprint System LDL Subfractions Kit; Quantimetrx, Redondo Beach, CA, USA).

Safety was assessed at every visit via patient reported AEs and regular monitoring of parameters including vital signs, physical exam, laboratory tests, and 12-lead electrocardiogram. Peripheral edema was defined as an increase of 10% or more in ankle circumference from baseline combined with pitting edema. As additional exploratory safety profiles, bone mineral density by dual-energy X-ray absorptiometry (DXA) and funduscopic examination by digital camera were also measured at the individual centers. An independent data safety monitoring board reviewed the safety data including liver dysfunction, weight gain and edema, anemia, heart failure, and cardiovascular events at 10%, 30%, 50%, 80% and 100% of study completion.

### Statistical analyses

Efficacy analysis was done on the full analysis set (the intention-to-treat population), comprising all randomized patients who received at least one dose of study medication and who had a baseline and at least one post-baseline efficacy measurement. The last-observation-carried-forward analysis was used to handle missing data, early discontinuation, or introduction of rescue therapy. All patients who received at least one dose of study medication were included in the safety analyses with descriptive statistics. This study data were collected using a paper CRF and entered into a computer database through double entry method. Entered data were validated by Data Validation System.

Data are expressed as mean ± SD for continuous variables, and data for the categorical variables are expressed as the number and the percentage of patients. Fisher's exact test or a chi-square test was used for categorical variables. Comparisons between groups were performed using Student's t-test or ANCOVA after adjusting baseline value, appropriately. The comparisons before and after treatment within groups were analyzed by a paired t test. A p value<0.05 was considered statistically significant. All the statistical analyses were performed using SAS version 9.2 (SAS Institute, Cary, NC).

A sample size of 117 patients (78 patients in the lobeglitazone group and 39 patients in the placebo group with a 2∶1 ratio) was needed to ensure 90% power to detect a difference of 1% between the two groups for HbA1c change from baseline to the end of the treatment period, assuming a common standard deviation of 1.57% [13], at a two-sided significance level of 0.05. Therefore, it was planned to randomize at least 168 patients (112 patients in the lobeglitazone group and 56 patients in the placebo group) to account for the 30% loss in follow-up. The protocol for this trial and CONSORT checklist are available as supporting information (Protocol S1 and Checklist S1). The study was registered with ClinicalTrials.gov (number NCT01001611).

## Results

### Study patients and baseline characteristics

The patients were enrolled between OCT 16^th^, 2009 and Oct 21^th^, 2010. Among the 252 patients screened, 173 patients were randomly assigned (a 2∶1 ratio) to lobeglitazone 0.5 mg (n = 115) or matching placebo (n = 58). The baseline demographic and clinical characteristics of study patients were comparable between groups (Table 1). All patients who received at least one dose of study medication were included in the safety analysis set (170 patients, lobeglitazone: n = 112; placebo: n = 58). Only two patients were excluded from the full efficacy analysis because of lack of post-baseline efficacy data. So, almost all of the patients were included in the efficacy analysis set (97.1% of randomized patients, lobeglitazone: n = 110; placebo: n = 58).

**Table 1:** Baseline demographic and clinical characteristics of the patients*.

	Lobeglitazone (n = 115)	Placebo (n = 58)	P value
Age (years)	56.36±9.28	54.72±9.70	0.2836^a^
Diabetes duration (years)	4.31±3.87	4.9±4.56	0.3797^a^
Male sex	66 (57.39%)	32 (55.17%)	0.7810^b^
BMI (kg/m^2^)	25.25±2.75	25.11±2.24	0.7408^a^
Weight (kg)	66.45±9.93	65.93±8.81	0.7353^a^
Waist circumference (cm)	88.18±8.03	86.94±5.78	0.2441^a^
Systolic blood pressure (mmHg)	124.17±13.35	123.31±12.23	0.6802^a^
Diastolic blood pressure (mmHg)	77.33±9.03	76.24±8.44	0.4453^a^
Fasting plasma glucose (mg/dL)	154.83±42.46	163.84±65.62	0.3448^a^
Hemoglobin A1c (%)	7.95±1.03	8.05±0.90	0.5132^a^
Total cholesterol (mg/dL)	179.63±32.58	188.26±37.66	0.1206^a^
LDL cholesterol (mg/dL)	110.24±33.17	114.76±34.01	0.4031^a^
Triglycerides (mg/dL)	137.03±74.07	177.14±119.34	0.0217^a^
HDL cholesterol (mg/dL)	48.78±12.73	46.33±13.56	0.2430^a^
Diabetes treatments			
Drug naïve	45 (39.13%)	24 (41.38%)	0.9317^b^
Stopped medication ≥3 months	23 (20.00%)	12 (20.69%)	
Stopped medication <3 months	47 (40.87%)	22 (37.93%)	

*Data are means (SD) or numbers (%).

BMI, body mass index; LDL, low-density lipoprotein; HDL, high-density lipoprotein.

a : P-values are for independent t-test.

b : P-values are for chi-square or Fisher's Exact test.

Overall, 83.2% of randomized patients (n = 144) completed the 24-week treatment period. The main reasons for discontinuation of treatment prematurely were withdrawal of consent and lack of efficacy and AEs. Loss to follow-up was similarly low in both treatment groups (withdrawal of consent (n = 7 vs 4), lack of efficacy (n = 5 vs 4), and AEs (n = 3 vs 0), in the lobeglitazone and the placebo groups, respectively). After further exclusion of patients due to protocol violation, poor compliance and rescue medication, 130 patients (75.1% of randomized patients) were included in the protocol analysis set **(Information S2)**.

### Efficacy

**Figure 1:**
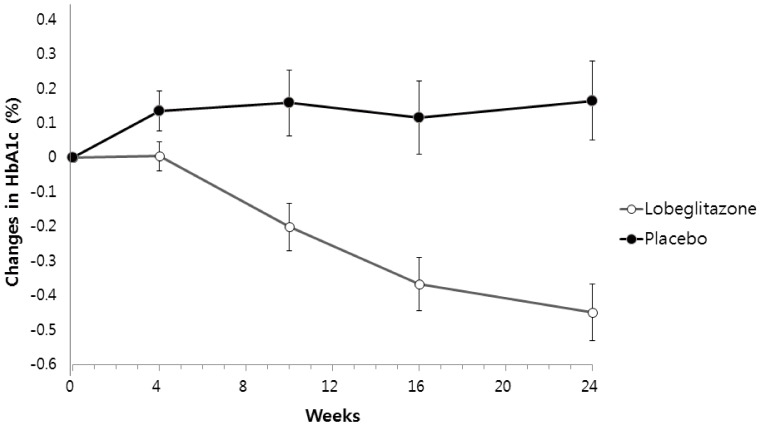
Adjusted mean changes (±SE) in HbA1c levels from baseline to week 24.

The predefined primary endpoint was the change in HbA1c from baseline to the end of treatment. Lobeglitazone significantly decreased HbA1c (from baseline 7.85±0.89% to study end 7.41±1.25%) compared to placebo (from baseline 8.05±0.9% to study end 8.21±1.12%), resulting in a mean treatment difference of −0.6% (p<0.0001). The baseline HbA1c adjusted mean difference between lobeglitazone and placebo was −0.62% (least square mean (SE), −0.45±0.08% vs 0.17±0.11%, p<0.0001, Figure 1). In the protocol set analysis, lobeglitazone also significantly decreased HbA1c by −0.57% (from 7.75±0.80% to 7.18±1.11%, p<0.0001), resulting in a mean treatment difference of −0.66% between the two groups (p<0.0001). The goal of HbA1c <7% was achieved significantly more in the lobeglitazone group compared to the placebo group (44% vs 12%, p<0.0001) in the efficacy analysis set (the intention-to-treat population).

FPG (p<0.0001), HOMA-IR (p = 0.002) and HOMA-β (p = 0.0277) were also improved in the lobeglitazone group, with no change observed in the placebo group (Table 2). In addition, lobeglitazone treatment significantly improved the levels of triglycerides, HDL-C, small dense LDL-C, free fatty acid, and Apo-B and Apo-CIII compared to placebo (all p<0.01, Table 2). The significant differences between groups were observed after 4 weeks of treatment for triglycerides, free fatty acid, and Apo-B and Apo-CIII, and after 10 weeks of treatment for HDL-C (data not presented). In addition, mean change in LDL-C was similar between groups, and no significant changes from baseline were recorded (Table 2).

**Table 2:** Effects on various gluco-metabolic and lipid parameters of lobeglitazone as compared with placebo from baseline to 24 weeks.

Variable	Lobeglitazone (n = 110)		Placebo (n = 58)		
	Baseline	Follow-up	Baseline	Follow-up	P value†
Fasting glucose (mg/dL)	150.91±37.51	131.80±37.39^c^	163.84±65.62	164.67±47.87	<.0001
Hemoglobin A1c (%)	7.85±0.89	7.41±1.25^c^	8.05±0.9	8.21±1.12	<.0001
Fasting insulin (µIU/mL)	9.30±3.76	8.55±3.69^a^	10.46±6.24	11.20±9.18	0.0298
HOMA-IR	3.51±1.86	2.80±1.58^c^	4.30±3.82	4.70±5.25	0.002
HOMA- β*	44.42±23.02	54.93±34.16^c^	44.48±26.65	45.05±35.89	0.0277
Total cholesterol (mg/dL)	178.70±32.08	184.70±34.48^a^	188.26±37.66	193.10±42.63	0.7191
Triglyceride (mg/dL)	137.51±74.72	118.45±56.24^b^	177.14±119.34	193.28±160.61	0.0006
HDL cholesterol (mg/dL)	48.69±12.78	52.99±13.62^c^	46.33±13.56	47.09±11.57	0.0038
LDL cholesterol (mg/dL)	109.00±32.25	109.95±32.89	114.76±34.01	112.12±29.83	0.6358
Small dense LDL (%)	8.10±6.70	6.40±6.55^a^	9.51±6.81	10.13±7.49	0.0033
Free Fatty acid (uEq/L)	622.28±214.18	561.89±236.24^a^	699.57±262.28	698.57±253.69	0.0047
Apolipoprotein AI (mg/dL)	150.06±26.29	152.01±25.24	147.98±29.50	151.91±24.41	0.6418
Apolipoprotein B (mg/dL)	80.15±19.64	76.01±18.73^b^	85.93±21.85	86.43±20.19	0.0046
Apolipoprotein CIII (mg/dL)	12.27±4.29	12.26±4.11	13.97±5.85	15.50±6.66^a^	0.0014

HOMA-β, homeostasis model assessment of β-cell function; HOMA-IR, homeostasis model assessment of insulin resistance; HDL, high-density lipoprotein; LDL, low-density lipoprotein;

* Outlier values are removed.

† P-values are for ANCOVA after adjusting baseline values. Multiple comparison by the Bonferroni was applied to analyses the primary endpoint (HbA1c).

a,b,c Significant changes from baseline to week 24 are indicated by ^a^ P<0.05; ^b^ P<0.01; ^c^ P<0.001 by paired t-test.

Last-observation-carried-forward analysis, the intention-to-treat population.

### Safety and tolerability

At 24 weeks, more weight gain was observed in the lobeglitazone group than in the placebo group (0.89 kg vs – 0.63 kg, mean difference 1.52 kg; p<0.0001). However, there were no significant differences in waist and ankle circumference between the two groups after treatment (p = 0.14 and 0.06, respectively). Systolic blood pressure, diastolic blood pressure, and pulse rate were not changed from baseline after treatment with lobeglitazone and were similar between the treatment groups (data not presented). In addition, there were no clinically significant changes in electrocardiogram after treatment with lobeglitazone.

The other safety profile was comparable between the two groups and lobeglitazone was also well tolerated (Table 3). The only AE considered drug-related that occurred at an incidence of ≥3% during the 24-week treatment period was peripheral edema (3.6% in the lobeglitazone group vs. 0% in the placebo group). No patient in this study had severe edema requiring discontinuation of the study drug. There were no differences between the lobeglitazone and placebo groups in the percentage of patients with increases of alanine aminotransferase, aspartate aminotransferase, total bilirubin, creatinine, creatine kinase, lactate dehydrogenase and N-terminal pro-brain natriuretic peptide (NT-pro BNP) during treatment. Funduscopic examination and bone mineral density measure were similar between the two groups during the treatment period (data not presented). Four patients in the lobeglitazone group experienced serious AEs. Serious AEs in the lobeglitazone 0.5 mg group included lung cancer, traumatic cerebral hemorrhage, cerebrovascular accident (underlying atrial fibrillation), and right scrotal laceration and hemorrhoidectomy. These serious AEs were not considered by the investigators to be related to the study medication. In addition, heart failure, ischemic heart disease, renal insufficiency, or bone fracture was not observed in both the two groups.

**Table 3:** Summary of clinical adverse events of the patients.

	Lobeglitazone (n = 112)	Placebo (n = 58)
**Any adverse event**	55 (49.1%)	30 (51.7%)
**Drug-related adverse event**	10 (8.9%)	3 (5.2%)
**Serious adverse event**	4 (3.6%)	0 (0%)
**Adverse event with frequency ≥3% in any group**		
Hyperglycemia†	3 (2.7%)	4 (6.9%)
Headache	3 (2.7%)	2 (3.5%)
Peripheral edema	4 (3.6%)	0 (0%)
Nasopharyngitis	6 (5.4%)	0 (0%)
Upper respiratory tract infection	2 (1.7%)	3 (5.2%)
Urticaria	0 (0%)	2 (3.5%)
Hematuria	3 (2.7%)	3 (5.2%)
Tingling sensation	0 (0%)	2 (3.5%)
**Adverse event of special interest**		
Heart failure	0 (0%)	0 (0%)
Ischemic heart disease	0 (0%)	0 (0%)
Anemia	2 (1.7%)	0 (0%)

Data are presented as n (%).

† Hyperglycemia is defined by prespecified criteria.

## Discussion

This study showed lobeglitazone monotherapy improved glycemic control in patients with T2DM inadequately controlled on diet and exercise. After 24 weeks, HbA1c as well as FPG were significantly decreased with lobeglitazone and the achievement rate of target (HbA1c <7%) was about 4-fold higher in the lobeglitazone group compared to the placebo group. The magnitude of improvement in HbA1c (placebo-subtracted change of −0.6%) was moderate in view of better effects observed in the *in vivo* and *in vitro* studies of lobeglitazone. We selected lobeglitazone 0.5 mg as the minimum effective dose to reduce the incidence of well-known AEs of TZDs, and this may be a reason to explain the modest glucose-lowering efficacy of lobeglitazone. Owing to a relationship between the dose and the AEs of TZDs, it could be a reasonable approach to use lower doses of TZDs in clinical practice. For instance, a Japanese study demonstrated the safety and efficacy of low-dose pioglitazone (7.5 mg/d), suggesting that it could be another good choice of treatment for T2DM [14].

Lobeglitazone monotherapy also produced improvements in the lipid parameters. For example, lobeglitazone treatment produced a 13% reduction from baseline triglycerides levels and an 8% rise from baseline HDL-C levels. Considering changes in the parameters observed with pioglitazone [1], the magnitude of improvements seemed to be similar or somewhat low. However, differently from rosiglitazone and sometimes pioglitazone, lobeglitazone did not increase LDL-C levels. In addition, significant improvements were observed in small dense LDL-C, free fatty acid, and Apo-B and Apo-CIII levels with lobeglitazone compared to placebo. The effects on lipids were recorded early after 4–10 weeks of lobeglitazone treatment. A phase III trial is underway in Korea to evaluate the effects of lobeglitazone 0.5 mg on glucose and lipid parameters compared to pioglitazone 15 mg in patients with T2DM.

Dyslipidemia in patients with T2DM is characterized by low levels of HDL-C and elevated triglyceride levels, associated with a higher proportion of small dense LDL particles [15], and the lipid levels are affected by glycemia or insulin resistance. Furthermore, hyperglycemia, dyslipidemia, and underlying insulin resistance are associated with increased risk of CVDs in T2DM. Thus, therapies targeting these gluco-metabolic abnormalities simultaneously, by modulating PPAR-γ and perhaps also the other PPARs, could be a reasonable strategy to prevent the future CVDs in diabetic patients. However, most of these drugs have been discontinued due to various safety concerns that have included increased cardiovascular risk (muraglitazar) [16], increase in plasma creatinine (tesaglitazar) [17], or liver toxicity and tumors in rodents (several earlier agents) [18]. The latest dual PPAR-α/γ agonist in development is aleglitazar (Hoffmann-La Roche), which is currently in the phase III trial to test the hypothesis that aleglitazar (1.5 mg daily dose) can reduce cardiovascular morbidity and mortality in patients with T2DM (NCT01042769).

Because many TZDs have issued various safety concerns, it is important to weigh efficacy and safety together in determining the clinical usefulness of novel TZDs. In the present study, lobeglitazone showed a good safety profile and well tolerated over the course of the 24-week. Weight gain and edema are well known AEs related to TZDs. Lobeglitazone treatment also increased body weight by 0.89 kg (placebo-subtracted mean difference: 1.52 kg) and was related to more peripheral edema (3.6%) compared to placebo. However, the magnitude of these AEs seems to be modest compared to other TZDs [1]. Furthermore, any heart failure was not observed during study period, although this study is too small and short. Also, the independent data safety monitoring board reviewed the safety data regularly and didn't find any drug related, serious AEs. The efficacy profile of lobeglitazone was similar to pioglitazone. Thus, a safety concern could be raised with respect to the risk of bladder cancer possibly related to pioglitazone [7]. However, a 2-year carcinogenicity study in rats treated with lobeglitazone showed no evidence of bladder cancer (data not presented); this result could be explained by the fact that lobeglitazone is mainly excreted by feces differently from pioglitazone.

Currently, the use of TZDs has decreased because of safety issues. Instead of them, new drugs (dipeptidyl peptidase-4 inhibitors, glucagon-like peptide-1 agonists and sodium-glucose co-transporter 2 inhibitors) are being welcomed by many clinician. However, none of these newer agents target insulin resistance. So, we believe that TZDs are a useful option for treating some diabetics especially in patients with insulin resistance – identified by an increased waist circumference, low HDL cholesterol or high triglyceride level, and non-alcoholic fatty liver disease.

Although lobeglitazone treatment improved hyperglycemia and the broad range of dyslipidemia, the sample size of this study was too small to make any definitive conclusion concerning clinical outcomes. There were no coronary events in this study. Thus, whether the observed favorable effects of lobeglitazone on various glucose and lipid parameters translates into actual benefits in terms of cardiovascular morbidity and mortality must await further investigation. Also, generalizability to non-Korean subjects of this study is uncertain because all of the subjects were Korean. Nevertheless, considering that subjects of our study were less obese and less insulin-resistant than Caucasians, we expect non-Korean, obese subjects may show larger HbA1c reduction compared to this study.

In conclusion, lobeglitazone 0.5 mg showed improvements in glucose and lipids endpoints with the favorable safety profile over 24 weeks. The results support a potential role of lobeglitazone in treating type 2 diabetes. A larger scale study with longer duration is needed to assess the long-term clinical benefit and risk of lobeglitazone.

## Supporting Information

Information S1 **Chemical structure of lobeglitazone (CKD-501).**
(TIF)

Information S2 **Trial profile (Enrollment, Randomization, and Follow-up of Study Patients).**
(TIF)

Checklist S1 **CONSORT checklist.**
(DOC)

Protocol S1 **Study protocol.**
(DOC)
