# Supplementary figures and images for: Efficacy and Safety of Lobeglitazone Monotherapy in Patients with Type 2 Diabetes Mellitus over 24-Weeks: A Multicenter, Randomized, Double-Blind, Parallel-Group, Placebo Controlled Trial
Source: PLoS One. 2014 Apr 15;9(4):e92843. doi: 10.1371/journal.pone.0092843 (PMC3988010; doi:10.1371/journal.pone.0092843)

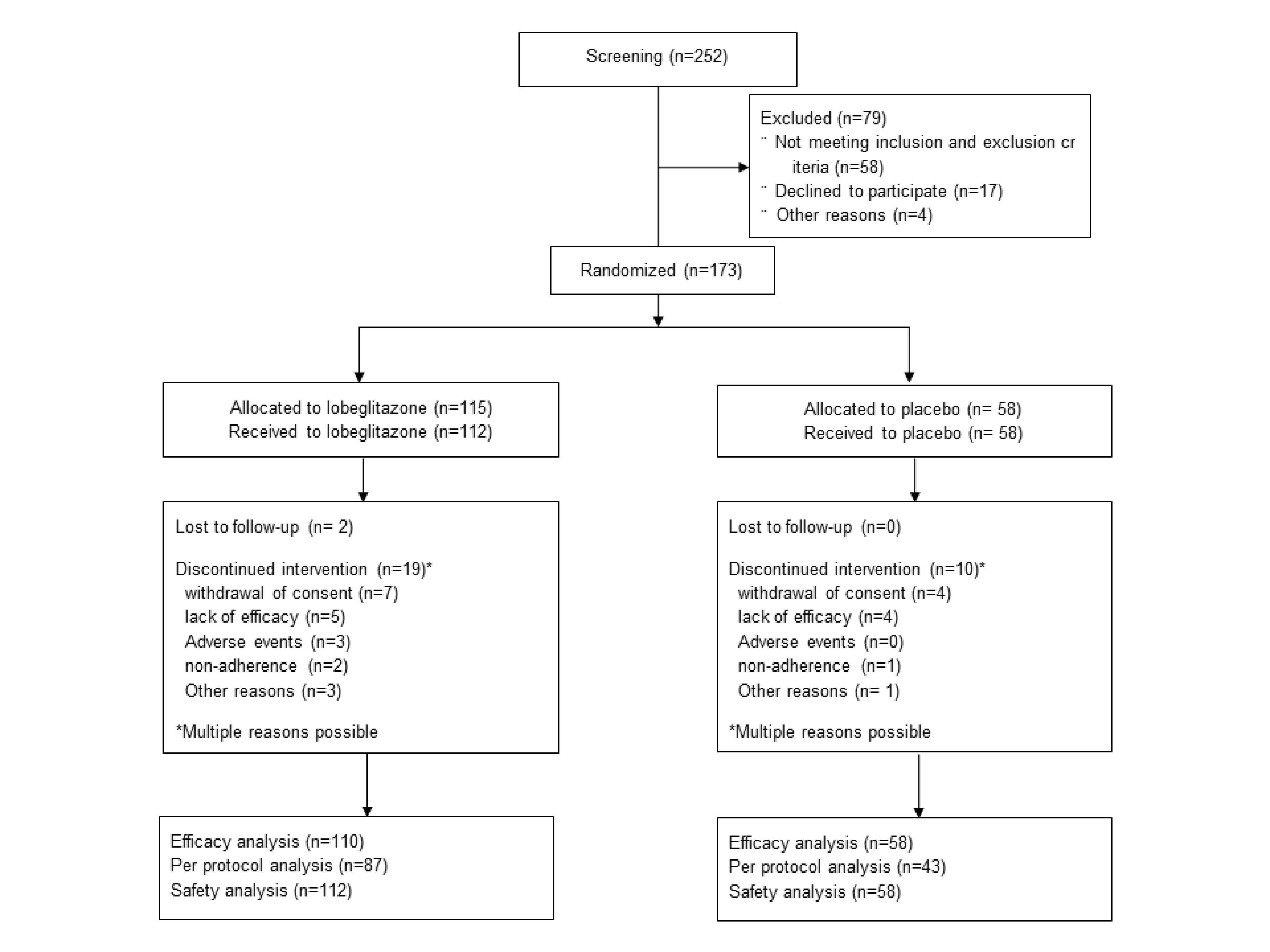

Supplement: Information S2 — Trial profile (Enrollment, Randomization, and Follow-up of Study Patients). (TIF) [file pone.0092843.s002.tif]
