# Supplementary material for: Efficacy and Safety of Lobeglitazone Monotherapy in Patients with Type 2 Diabetes Mellitus over 24-Weeks: A Multicenter, Randomized, Double-Blind, Parallel-Group, Placebo Controlled Trial
Source: PLoS One. 2014 Apr 15;9(4):e92843. doi: 10.1371/journal.pone.0092843 (PMC3988010; doi:10.1371/journal.pone.0092843)
Supplement: Protocol S1 — Study protocol. (DOC) [file pone.0092843.s004.doc]

**Synopsis**

| **Title** | An Evaluation of Glycemic control effects of Monotherapy CKD-501 in Patients With Type 2 Diabetes Mellitus: A 24-week, multi center, randomized, double-blind, parallel-group, placebo control, therapeutic confirmatory study. |
| --- | --- |
| **Background** | Thiazolidinediones (TZDs), peroxisome proliferator-activated receptor (PPAR)-γ agonists, are the first drugs that improve insulin sensitivity in skeletal muscle and reduce hepatic glucose production in patients with type 2 diabetes mellitus (T2DM). They do not increase the risk of hypoglycemia and are more durable in controlling hyperglycemia than sulfonylureas and metformin. Furthermore, pioglitazone has a beneficial effect on the lipid profile in patients with T2DM. The PROspective pioglitAzone Clinical Trial In macroVascular Events (PROactive) trial showed a benefit on major cardiovascular events as a secondary outcome in patients with a prior cardiovascular event or with multiple risk factors for cardiovascular disease (CVD).  However, TZDs may have clinically significant adverse effects (AEs), such as body-weight gain, fluid retention, congestive heart failure, bone fractures, increased risk of myocardial infarction. Therefore, there is a need to develop more effective and safe antidiabetic drugs targeting PPAR-γ.  Lobeglitazone (CKD-501; Chong Kun Dang Pharmaceutical Corp., Seoul, Korea) is a novel PPAR-γ agonist with substituted pyrimidine derivatives containing TZD. Lobeglitazone showed more potent activity than the reference compounds (i.e. pioglitazone and rosiglitazone) in both in vitro and in vivo studies. Therefore, lobeglitazone is expected to improve insulin sensitivity, and glucose and blood lipid profiles with a lower effective dose. |
| **Objectives**  **(Endpoints)** | ► **Primary objective**  To compare the change from baseline in HbA1c achieved with each dose of CKD-501 versus placebo in treatment subjects with type 2 diabetes who have inadequate glycemic control on exercise and diet.  ► **Secondary objectives**   1. To evaluate the glycemic parameter (Fasting plasma glucose, C-peptide, HOMA-IR, HOMA-β, QUICKI) change with CKD-501 0.5mg versus placebo after 24 weeks of treatment 2. To evaluate the proportion of participants achieving a therapeutic response (HbA1c < 7%) with CKD-501 0.5mg versus placebo after 24 weeks of treatment 3. To evaluate the lipid parameters(Total Cholesterol, Triglycerides, LDL–C, HDL-C, Small Dense LDL–C, FFA, Apo-AⅠ/B/ CⅢ) change with with CKD-501 0.5mg versus placebo after 24 weeks of treatment 4. To assess safety and tolerability (physical examination, vital sign, laboratory evaluation, ECG and adverse events) of CKD-501 0.5mg after 24 weeks of treatment 5. To explore the safety and tolerability of CKD-501 0.5mg after 52 weeks of treatment |
| **Study Chair** | Dong Seop Choi, MD, PHD.  Division of Endocrinology and Metabolism, Department of Internal Medicine, Korea University Anam Hospital. |
| **Investigators** | | **Site (Korea, Republic)** | **Investigator** | | --- | --- | | Division of Endocrinology and Metabolism, Department of Internal Medicine, Korea University Anam Hospital. | Dong Seop Choi,  MD, PHD. | | Division of Endocrinology and Metabolism, Department of Internal Medicine, Hallym University Kangdong Sacred Heart Hospital | Doo-Man Kim, MD, PHD. | | Division of Endocrinology and Metabolism, Department of Internal Medicine, Kyung Hee University Hospital | Jeong-Taek Woo, MD, PHD. | | Division of Endocrinology and Metabolism, Department of Internal Medicine, Seoul National University Bundang Hospital | Hak Chul Jang, MD, PHD. | | Division of Endocrinology and Metabolism, Department of Internal Medicine, Wonju Severance Christian Hospital | Choon Hee Chung, MD, PHD. | | Division of Endocrinology and Metabolism, Department of Internal Medicine, Inje University Sanggye Paik Hospital | Kyung Soo Ko,  MD, PHD. | | Division of Endocrinology and Metabolism, Department of Internal Medicine, Inje University Pusan Paik Hospital | Jeong Hyun Park, MD, PHD. | | Division of Endocrinology and Metabolism, Department of Internal Medicine, Hanyang University Guri Hospital | Yong Soo Park, MD, PHD. | | Division of Endocrinology and Metabolism, Department of Internal Medicine, Soon Chun Hyang University Cheonan Hospital | Sang Jin Kim,  MD, PHD. | |
| **Sponsor** | Chong Kun Dang Pharmaceutical corp. |
| **Target Disease** | Type 2 Diabetes mellitus |
| **Eligibility** | ► Inclusion Criteria:   1. Males or Females with type 2 diabetes 2. Diagnosis of T2DM for at least 3 months prior to study entry (Visit 1) 3. Age in the range of 18-80 years inclusive 4. At visit 1, 5. Current use an OHA or not on an OHA < 3 months: HbA1c 6.5~9% 6. Drug naïve or not on an OHA ≥ 3 months : HbA1c 7-10% 7. Body mass index(BMI) in the range of 21-40 kg/m^2^ (Visit 1) 8. C-peptide > 1.0ng/ml (Visit 1) 9. Women to be: 10. Surgical sterile 11. Postmenopausal women 45 years of age or older   Postmenopausal women is defined as: last menstruation > 24 months prior to signing informed conscent)   1. A female of childbearing potential using a acceptable methods of birth control until about 14days after the last dose.   Two or more(including barrier method) the use of contraception.   1. Male using an acceptable method on birth control. 2. Written informed consent to participate in the study   ► Exclusion criteria   1. Type 1 diabetes mellitus, gestational Diabetes or secondary form of diabetes 2. Treatment with insulin or thiazolidinediones within 60 days prior to visit 1 3. Fasting plasma glucose > 250mg/dL (Visit 1) 4. Tryglyceride ≥ 500mg/dL (Visit 1) 5. Uncontrollable hypertension(Although treat with antihypertension agent, systolic blood pressure greater than 140 mmHg and diastolic blood pressure greater than 90 mmHg) 6. History of myocardial infarction, heart failure(NYHA Class III~IV), cerebral infarction, hematencephalon or unstable angina within 6 months  | **NYHA Class** | **Symptoms** | | --- | --- | | I | Cardiac disease, but no symptoms and no limitation in ordinary physical activity, e.g. shortness of breath when walking, climbing stairs etc. | | II | Mild symptoms (mild shortness of breath and/or angina) and slight limitation during ordinary activity. | | III | Marked limitation in activity due to symptoms, even during less-than-ordinary activity, e.g. walking short distances (20–100 m). Comfortable only at rest. | | IV | Severe limitations. Experiences symptoms even while *at rest*. Mostly bedbound patients. |  1. Severe liver or renal dysfunction:   - liver dysfunction: ALT, AST, Total bilirubin, Alkaline Phosphatase level greater than 2.5 times the upper limit of the normal range (visit 1)  - renal dysfunction: renal failure or serum creatinine greater than 30% the upper limit of the normal range (visit 1)   1. For any reason, patients with anemia:   Hb ≤ 11.5.g/dL males, Hb ≤ 10.5g/dL females (visit 1)   1. Systemic or inhalation corticosteroids treatment within 1 months prior to visit 1 2. Needs treatment for acute disease, uncontrolled other diseae or diabetic complications 3. Patients with thyroid dysfunction(out of normal TSH range) 4. History of proliferative diabetic retinopathy 5. In treatment concomitant drug having severe risk drug interaction with investigational drug 6. History of cancer within 5 years 7. History of active substance abuse including alcohol 8. Hepatitis B Antigen(HBsAg) test is positive (visit 1) 9. Patient who have experience such as hypersensitivity reaction, serious adverse event or no effect by treatment with glitazones 10. Fertile women who not practice contraception with appropriate methods 11. Pregnant or lacting female 12. Has a contraindication to treatment investigational drug from the medical and psychogenic side 13. An impossible one who participates in clinical trial by legal 14. Any investigational drug therapy within 4 weeks of signing the informed consent   23) In the opinion of the investigator, unable to comply with the requirements of the study protocol or unsuitable for the study for any reason |
| **Number of Subjects** | 168 randomized patients (CKD-501 group 112 patients, Placebo group 56 patients)  ► A sample size of 117 patients (78 patients in the lobeglitazone group and 39 patients in the placebo group with a 2:1 ratio) was needed to ensure 90% power to detect a difference of 1% between the two groups for HbA1c change from baseline to the end of the treatment period, assuming a common standard deviation of 1.57%, at a two-sided significance level of 0.05. Therefore, it was planned to randomize at least 168 patients (112 patients in the lobeglitazone group and 56 patients in the placebo group) to account for the 30% loss in follow-up. |
| **Investigational Product** | ► Investigator drug:  CKD-501 0.5mg  ► Comparator drug:  Placebo |
| **Design** | multi center, randomized, double-blind, parallel-group, placebo control study |
| **Investigational product dose** | ► Run in period  Subjects will administer placebo, orally, 1 tablet once daily for 2 weeks. (single-blind)  ► Treatment period  Subjects will be assigned to double-blind treatment with one of the 2 treatment regimens below in a ratio of 2:1;   - CKD-501 0.5mg , orally, 1 tablet once daily for 24 weeks - Placebo, orally, 1 tablet once daily for 24 weeks |
| **Methods** | Patients willing to paricipate will be screened after written informed consent is abtained.  Wash-out & placebo run-in period:   1. Current use an OHA or not on an OHA < 3 months (HbA1c 6.5~9%):   Patients enter a 4 week wash-out period.  After the wash-out period, patients enter a 2 week single-blind placebo run-in period.   1. Drug naïve or not on an OHA ≥ 3 months (HbA1c 7-10%):   Patients directly enter a 2 week single-blind placebo run-in period.  Following a 2 week single blind placebo run-in period, eligible subjects were randomized in a 2:1 ratio to receive 24 weeks of double-blind study medication: CKD-501 0.5mg (1 tablet qd) or placebo (1 tablet qd)  Patients who have completed the full 24 week period of trial are eligible for participation in the open-label extension study. All participants in the extension study will receive a 0.5mg once daily oral dose of CKD-501 for an additional 28 weeks.  Rescue treatment(Metformin) could be started if the participant had a FPG > 270mg/dl at 4, 10 week, FPG > 240mg/dl at 16, 24 week, FPG > 200mg/dl at 28, 40 week. |
| **Statistical**  **analysis** | Efficacy analysis was done on the full analysis set (the intention-to-treat population), comprising all randomized patients who received at least one dose of study medication and who had a baseline and at least one post-baseline efficacy measurement. The last-observation-carried-forward analysis was used to handle missing data, early discontinuation, or introduction of rescue therapy. All patients who received at least one dose of study medication were included in the safety analyses with descriptive statistics.  ► Demographic information and baseline characteristics   - For the comparison of demographic information and other baseline characteristics, continuous variables are analyzed using the independence-sample t test or ANCOVA after adjusting baseline value, appropriately and categorical variables are analyzed by using chi-square test or Fisher's exact test.   ► Efficacy  Primary endpoint   - The primary efficacy endpoint is the change in mean HbA1c from baseline (at randomization) at 24 weeks of CKD-501 0.5mg or placebo treatment. Differences between treatment groups are determined using independence-sample t test. If the CKD-501 0.5mg is statistically significant decrease compared to placebo, CKD-501 0.5mg is to demonstrate the superiority.   Secondary endpoint   - The secondary endpoint is the mean changes from baseline to 24 weeks in Glycemic parameters(Fasting plasma glucose, C-peptide, HOMA-IR, HOMA-β, QUICKI), Lipid Parameters(Total Cholesterol, Triglycerides, LDL–C, HDL-C, Small Dense LDL–C, FFA, Apo-AⅠ/B/ CⅢ Glycemic parameters and Lipid Parameters are analyzed by using a paired t-test in each treatment group. Between-group differences in these changes are analyzed by using independence samples t-test. - The proportion of subjects achieving their HbA1c level goal (HbA1c<7%) at week 24 is estimated according to treatment group, and a chi-square test or fisher’s exact test be performed to evaluate between-group differences.   ► Safety   - Changes from baseline in vital sign and physical examination at 24 weeks are analyzed by using a paired t-test in each treatment group. Between-group differences in these changes are analyzed by using independence samples t-test. - Each visit by a laboratory test results mean, standard deviation, as described by descriptive statistics and changes from baseline at 24 weeks are analyzed by using a paired t-test in each treatment group. Between-group differences in these changes are analyzed by using independence samples t-test.   During the study period, treatment-emergent adverse events was classified by System Organ Class(SOC) and types, following WHO-ART, and severity of adverse events, causal relation to the study drug are analyzed. Also, the incidence rate of every AE that occurred during the study period, chi-squared test or fisher’s exact test is used. |

**Flow chart**

| Day  Action | Screening Period^1)^ | Run-in period | Treatment period | | | | | Extension period | | |
| --- | --- | --- | --- | --- | --- | --- | --- | --- | --- | --- |
|  | V1 | V2 | V3 | V4 | V5 | V6 | V7 | E-V1 | E-V2 | E-V3 |
|  | -70d(-10wk)~-42d(-6wk) | -14d(-2wk) | 1d | 28d(4wk) | 70d(10wk) | 112d(16wk) | 168d(24wk) | 196d(28wk) | 280d(40wk) | 364d(52wk) |
| Visit | **X** | **X** | **X** | **X** | **X** | **X** | **X** | **X** | **X** | **X** |
| Written informed consent | **X** |  |  |  |  |  | **X** |  |  |  |
| Screening Number | **X** |  |  |  |  |  |  |  |  |  |
| Medical history & Demographics | **X** |  |  |  |  |  |  |  |  |  |
| Diet & Exercise training | **X** | **X** | **X** | **X** | **X** | **X** | **X** | **X** | **X** | **X** |
| V/S, Physical examination | **X** | **X** | **X** | **X** | **X** | **X** | **X** | **X** | **X** | **X** |
| Laboratory assesments including serum or urine hcg | **X** |  | **X** | **X** | **X** | **X** | **X** | **X** | **X** | **X** |
| ECG | **X** |  |  |  | **X** |  | **X** |  | **X** | **X** |
| fundus photo, BMD | **X** |  |  |  |  |  | **X** |  |  | **X** |
| HbA1c | **X** |  | **X** | **X** | **X** | **X** | **X** | **X** | **X** | **X** |
| Glycemic parameters | **X** |  | **X** | **X** | **X** | **X** | **X** | **X** | **X** | **X** |
| Lipid parameters | **X** |  | **X** | **X** | **X** | **X** | **X** | **X** | **X** | **X** |
| Review Inclusion/Exclusion criteria | **X** |  |  |  |  |  |  |  |  |  |
| Allocation No.(Randominization) |  |  | **X** |  |  |  |  |  |  |  |
| Administer study drug |  | **X** | **X** | **X** | **X** | **X** | **X** | **X** | **X** |  |
| Assess study drug compiance |  |  | **X** | **X** | **X** | **X** | **X** | **X** | **X** | **X** |
| Record concomitant | **X** | **X** | **X** | **X** | **X** | **X** | **X** | **X** | **X** | **X** |
| Safety evaluation |  | **X** | **X** | **X** | **X** | **X** | **X** | **X** | **X** | **X** |
